# PROVIDERS AND DIRECT CARE WORKERS’ PERCEPTIONS OF RETENTION

**DOI:** 10.1093/geroni/igad104.0976

**Published:** 2023-12-21

**Authors:** Isha Karmacharya, Leah Janssen, Bailee Brekke

**Affiliations:** Miami University, Oxford, Ohio, United States; Scripps Gerontology Center / Miami University, Oxford, Ohio, United States; Miami University, Oxford, Ohio, United States

## Abstract

Direct care worker (DCW) retention remains a persistent challenge for long-term services and supports (LTSS). Multiple studies have identified key factors for worker retention, including worker training and empowerment, career advancement opportunities, compensation, and communication. However, few studies include multiple perspectives on understanding work culture from both providers and DCWs, which is essential for developing effective retention strategies that address the needs and concerns of different stakeholders. This qualitative study explores elements of work culture through semi-structured in-depth interviews with 21 providers and 16 DCWs from 12 high-performing LTSS facilities and agencies across Ohio. The facilities/agencies were selected based on state-level family satisfaction and staff retention data. Three major elements of work culture emerged as important for DCW retention: 1) a family-like approach, 2) working conditions, and 3) worker empowerment. DCWs value a family-like approach in the workplace, which included relationships with clients and their supervisors. Favorable working conditions comprised freedom of choice, participative leadership, and effective communication that promoted retention. DCWs experienced empowerment through recognition and appreciation, coupled with financial incentives and career advancement opportunities. These findings suggest simple modifiable changes to work culture that improve worker retention in LTSS. This study urges policymakers to consider these implications for addressing the ongoing challenge of DCW retention.

